# Supplementary material for: Congenital SARS-CoV-2 Infection in Two Neonates with Confirmation by Viral Culture of the Placenta in One Case
Source: Viruses. 2023 May 31;15(6):1310. doi: 10.3390/v15061310 (PMC10301936; doi:10.3390/v15061310)

Supplementary Materials:

The following supporting information can be downloaded at: https://www.mdpi.com/article/10.3390/v15061310/s1, Table S1: Case 1 and Case 2 Summary of Selected Investigations, Figure S1: Selected Portion of Cardiotocographs Case 1 and Case 2.

Table S1 – Case 1 and Case 2 – Summary of Selected Investigations

CASE 1	CASE 2
Day 1<br>CRP – 0.8 mg/L<br>CBC – Hemoglobin 110 g/L, Platelets 253 X 10^9/L, WBC 6.4 X 10^9/L (neutrophils 1.5 X 10^9/L, lymphocytes 4.7 X 10^9/L)<br><br>Blood culture – No Growth<br>Bilirubin 77 μmol/L<br><br>Chest radiograph - Lung volumes are mildly diminished. Lung and pleural spaces are clear.	Day 1<br>CRP < 3 mg/L<br>CBC – Hemoglobin 165 g/L, Platelets 196 X 10^9/L, WBC 20 X 10^9/L (neutrophils 14.8 X 10^9/L, lymphocytes 4.0 X 10^9/L)<br><br>Blood culture – No Growth<br>Bilirubin total 129 umol/L<br><br>Arterial Cord Blood Gas<br>Ph 7.33, PCO2 36 mm Hg, pO2 24 mmg Hg<br><br>Venous Cord Blood Gas<br>pH 7.32, pCO2 43 mm Hg, pO2 23 mm Hg,<br><br>Chest radiograph - fine interstitial pattern in both lungs
Day 2<br>Bilirubin 143 umol/L	Day 2<br>CRP < 3 mg/L<br>Bilirubin 191 umol/L<br>Creatinine 29 umol/L<br><br>pH Venous Blood gas<br>pH 7.33, pCO2 38 mm Hg, pO2 51 mm Hg<br>lactate 1.6 mmol/L<br><br>Chest radiograph – appearance of lungs improved compared to prior exam
Day 5<br>CRP – 1.0 mg/L<br>CBC - Hemoglobin 117 g/L, Platelets of 177 X 10^9/L, WBC 5.0 X 10^9/L (neutrophils 0.8 X 10^9/L, lymphocytes 3.4 X 10^9/L)<br>Bilirubin 163 umol/L<br><br>Capillary blood gas: pH 7.38, pCO2 39 mmHg, pO2 36 mmHg, lactate 2.7 mmol/L, and glucose 6.1 mmol/L<br><br>Chest Radiograph - No infiltration or air leak<br>Blood culture – No Growth	Day 5<br>Bilirubin 250 umol/L

<u>Cerebrospinal fluid</u><br>Red Blood Cells 398,000 X 10^6/L<br>White Blood Cells 1112 X 10^6/L WBC (14% neutrophils)<br>NEGATIVE PCR for Herpes simplex virus (HSV 1 & 2), Varicella zoster virus, Enterovirus, Parechovirus<br>Culture showed one colony of *Micrococcus* species which was a contaminant	
<u>Day 6</u><br>Cranial ultrasound - right-sided germinal matrix hemorrhage (resolved by 5 weeks of age)<br><br>Chest radiograph - Slight increased lung markings bilaterally consistent with interstitial fluid and or early RDS	<u>Day 6</u><br>Creatinine 13 umol/L, Urea 7.0 mmol/L<br>Calcium 2.45 mmol/L, Magnesium 0.93 mmol/L<br>Sodium 138 mmol/L, Chloride 111 mmol/L, Phosphate 2.09 mmol/L, Albumin 32 g/L
<u>Day 7</u><br><u>Cerebrospinal fluid</u><br>Red Blood Cells 77,000 X 10^6/L<br>White Blood Cells 671 X 10^6/L WBC (42% neutrophils)	
<u>Day 8</u><br>CRP 1.6 mg/L	<u>Day 8</u><br>CRP < 3 mg/L<br>Troponin I Cardiac 0.06 ug/L)<br>CBC – Hemoglobin 176 g/L, Platelets 234 X 10^9/L, WBC 13.1 X 10^9/L (neutrophils 3.5 X 10^9/L, lymphocytes 7.5 X 10^9/L)
<u>Week 5</u><br>Cranial ultrasound - new left sided caudothalamic groove hemorrhage (resolved by 4 months of age)	

Figure S1 – Selected Portion of Cardiotocographs Case 1 and Case 2

Case 1 – Repetitive decelerations

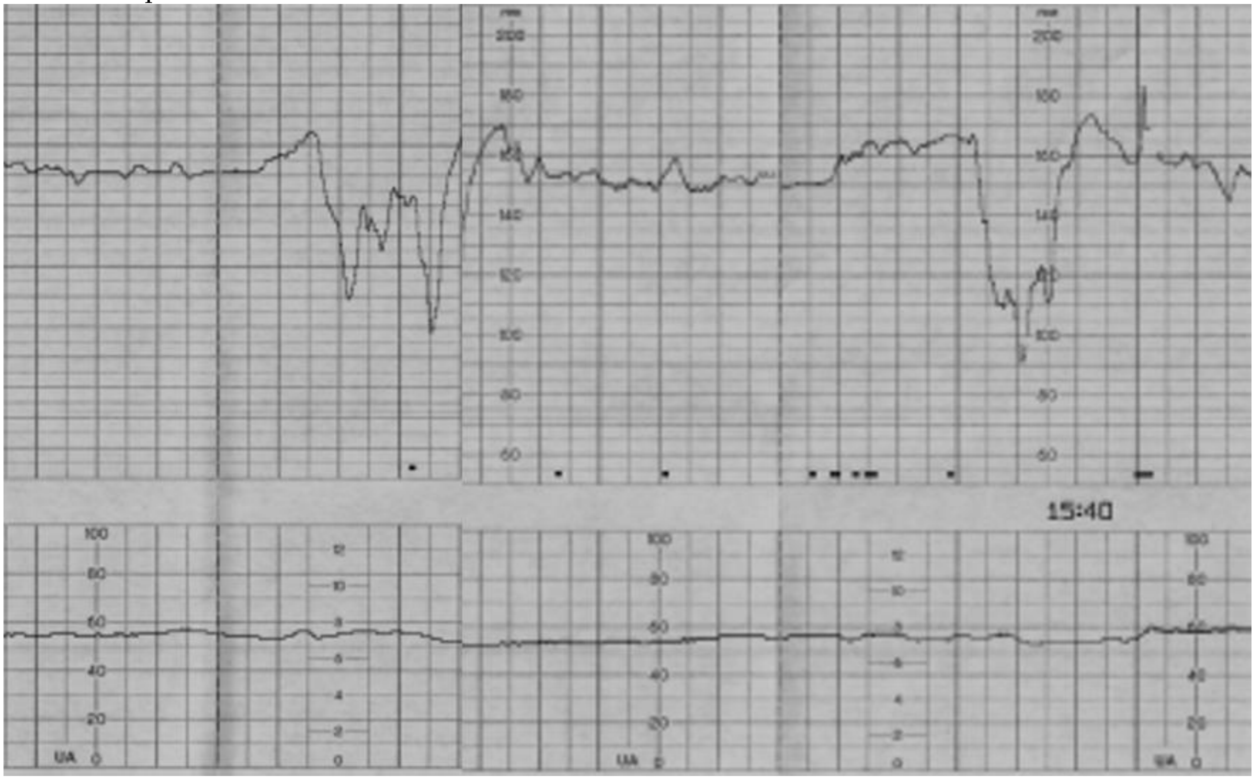

Case 2 – Fetal tachycardia with repetitive decelerations

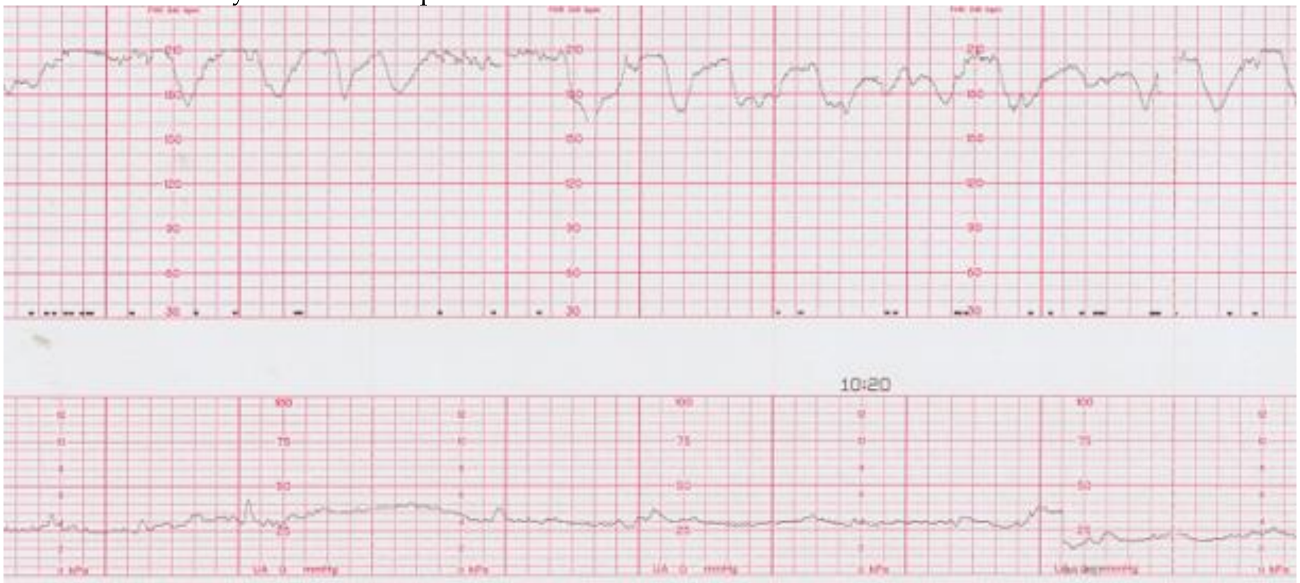

Supplement: Supplementary file 1 [file viruses-15-01310-s001.zip › viruses-2267686-supplementary.pdf]
